# Lung cancer recruitment of CD14+ cells results in an immunosuppressive phenotype and improved tumor survival under stress conditions

**DOI:** 10.1186/2051-1426-3-S2-P416

**Published:** 2015-11-04

**Authors:** Erin Schenk, Allan Dietz

**Affiliations:** 1 Mayo Clinic, Rochester, MN, USA

## 

We have previously shown that a higher burden of CD14+HLA-DR^lo/neg^ cells in the peripheral blood of cancer patients is associated with systemic immune suppression and a poor outcome. We have also reported that CD14+ cells isolated from healthy donors are converted to the immunosuppressive CD14+HLA-DR^lo/neg^ phenotype when cocultured with tumor cell lines or primary tumor cultures. Therefore, one potential mechanism of CD14+HLA-DR^lo/neg^ generation in cancer patients is the recruitment of CD14+ cells to the tumor microenvironment. In this study, we tested the ability of multiple human lung cancer cell lines to recruit CD14+ cells, convert normal donor CD14+ cells to CD14+HLA-DR^lo/neg^, and the impact on tumor survival under stress conditions in the presence of CD14+HLA-DR^lo/neg^ cells. Monocyte recruitment by cell line supernatants was studied using freshly isolated CD14+ cells and measured in real time with live cell imaging. Monocytes from 10/10 unique healthy donors migrated towards H23 supernatant in a dose dependent fashion. Supernatants from other human lung cancer cell lines including H889, H1155, H1581, and H1975 also recruited healthy donor monocytes 6 to 9 fold greater than culture media alone. The CD14+ phenotype resulting from interaction with lung cancer cell lines was studied in a 2 day coculture system. Coculture of H1581 with monocytes from 27 of 29 unique healthy donors resulted in a downregulation of monocyte cell surface expression of HLA-DR by 48.7% ± 22.1%, a phenotype known to mediate both local and systemic immune suppression. C14+ HLA-DR downregulation after coculture was observed with H23 (17/18 unique healthy donors, 49.7% ± 22.1% HLA-DR reduction) and H889 (5/6 unique healthy donors, 47.3% ± 16.1% HLA-DR reduction). Importantly, CD14+ cells promoted lung tumor cell line survival under stress conditions. After 6 days of low serum growth conditions, the survival and proliferation of H23 and H1581 was approximately 2 fold greater when cocultured with CD14+ cells than without monocytes. H889 continuously exposed to cisplatin in culture had significantly improved survival when co-cultured with monocytes for 2 days prior to cisplatin exposure. In conclusion, we have demonstrated multiple lung cancer cell lines recruit and transformation monocytes to the HLA-DR^lo/neg^ phenotype. These data suggest monocyte cancer cross-talk results in improved tumor survival when exposed to the likely *in vivo* conditions of a nutrient poor environment or chemotherapy independent of local immune suppression and angiogenesis.

